# STAR_outliers: a python package that separates univariate outliers from non-normal distributions

**DOI:** 10.1186/s13040-023-00342-0

**Published:** 2023-09-04

**Authors:** John T. Gregg, Jason H. Moore

**Affiliations:** 1 Department of Biostatistics, Epidemiology and Informatics, University of Pennsylvania, Philadelphia, PA USA; 2 Department of Computational Biomedicine, Cedars-Sinai Medical Center, Los Angeles, CA 90069 USA

**Keywords:** Outliers, Statistics, Software

## Abstract

There are not currently any univariate outlier detection algorithms that transform and model arbitrarily shaped distributions to remove univariate outliers. Some algorithms model skew, even fewer model kurtosis, and none of them model bimodality and monotonicity. To overcome these challenges, we have implemented an algorithm for Skew and Tail-heaviness Adjusted Removal of Outliers (STAR_outliers) that robustly removes univariate outliers from distributions with many different shape profiles, including extreme skew, extreme kurtosis, bimodality, and monotonicity. We show that STAR_outliers removes simulated outliers with greater recall and precision than several general algorithms, and it also models the outlier bounds of real data distributions with greater accuracy.

**Background** Reliably removing univariate outliers from arbitrarily shaped distributions is a difficult task. Incorrectly assuming unimodality or overestimating tail heaviness fails to remove outliers, while underestimating tail heaviness incorrectly removes regular data from the tails. Skew often produces one heavy tail and one light tail, and we show that several sophisticated outlier removal algorithms often fail to remove outliers from the light tail. Multivariate outlier detection algorithms have recently become popular, but having tested PyOD’s multivariate outlier removal algorithms, we found them to be inadequate for univariate outlier removal. They usually do not allow for univariate input, and they do not fit their distributions of outliership scores with a model on which an outlier threshold can be accurately established. Thus, there is a need for a flexible outlier removal algorithm that can model arbitrarily shaped univariate distributions.

**Results** In order to effectively model arbitrarily shaped univariate distributions, we have combined several well-established algorithms into a new algorithm called STAR_outliers. STAR_outliers removes more simulated true outliers and fewer non-outliers than several other univariate algorithms. These include several normality-assuming outlier removal methods, PyOD’s isolation forest (IF) outlier removal algorithm (ACM Transactions on Knowledge Discovery from Data (TKDD) 6:3, 2012) with default settings, and an IQR based algorithm by Verardi and Vermandele that removes outliers while accounting for skew and kurtosis (Verardi and Vermandele, Journal de la Société Française de Statistique 157:90–114, 2016). Since the IF algorithm’s default model poorly fit the outliership scores, we also compared the isolation forest algorithm with a model that entails removing as many datapoints as STAR_outliers does in order of decreasing outliership scores. We also compared these algorithms on the publicly available 2018 National Health and Nutrition Examination Survey (NHANES) data by setting the outlier threshold to keep values falling within the main 99.3 percent of the fitted model’s domain. We show that our STAR_outliers algorithm removes significantly closer to 0.7 percent of values from these features than other outlier removal methods on average.

**Conclusions** STAR_outliers is an easily implemented python package for removing outliers that outperforms multiple commonly used methods of univariate outlier removal.

**Supplementary Information:**

The online version contains supplementary material available at 10.1186/s13040-023-00342-0.

## Background

### Outlier removal as a process

Outliers are defined as datapoints that 1) arise from a stochastic process that the researcher does not want to measure and 2) reside discernibly far from the body of the main distribution [1]. Researchers tend to assume that (1) is true if (2) is true, the idea being that such rare and extreme values distort parameter estimates if they are true outliers while contributing relatively little otherwise [1]. The process of removing outliers includes two steps. The first step is to transform the data, sometimes referring to the transformed values as outlier scores. The second step is one of two options. The first option is to label known outliers and train a model to predict outlier status from the outlier scores, which is beyond the scope of this paper because it requires knowing outlier statuses for a subset of the data.

The second option, which this paper will consider, is to fit a distributional model to those outlier scores and remove scores beyond a certain percentile of a fitted model. Assuming that non-outliers in the data follow the chosen model, measured values beyond a certain percentile of the fitted model are removed from the analysis because they are especially likely to be outliers. Researchers sometimes remove the outermost percentiles of outlier scores instead of using a fitted model’s percentile threshold, but such data truncation introduces bias into downstream analyses. For this reason, our results primarily analyze existing outlier detection methods that specifically remove outliership scores beyond some threshold of a fitted model, though we also review the benefits and drawbacks of PyOD’s multivariate outlier removal algorithms.

### Nonparametric univariate outlier removal methods

Nonparametric outlier detection methods compute outliership scores without using a parametric model, though the distribution of outliership scores usually still needs to be fitted with a model to provide the reference quantile cutoff. This may be the reason that we could find few complete nonparametric outlier removal algorithms with established code. Unlike the majority of algorithms in the PyOD library [2], the IF method does contain a default model to estimate datapoints’ univariate outliership statuses [3]. Briefly, isolation forests repeatedly split the distribution into subsets at randomly chosen domain values and count how many splits are required to isolate each point. Points that require fewer splits to isolate are more likely to be outliers. The number of splits required for this to occur, or possibly some function of the output from several repetitions of this procedure, comprises the outliership score.

### Multivariate outlier removal methods

In general, multivariate outlier detection methods search for vector values that are, by some multivariate metric, atypically distant from their distributions. To summarize a few examples, consider n random vector variables $\{{X}_{i} \forall i\in [N]\}$, where ${{X}_{i}}^{j}$ is the jth scalar component of the ith vector variable. ECOD [4] and COPOD [5] both define their outliership scores with a distance metric of $-{\sum }_{j}{log(F}^{j }({{X}_{i}}^{j})),$ where ${F}^{j}$ is the probability of observing a scalar value more extreme than that of ${{X}_{i}}^{j}$. Instead of considering the explicit distance of ${X}_{i}$ from the other datapoints, ABOD’s [6] outliership score computes the variance of all inverse distance weighted angles formed with ${X}_{i}$ at the apex. This metric is sensible because three point angles with ${X}_{i}$ at the apex tend to be smaller when ${X}_{i}$ is farther from the distribution, which corresponds to smaller variance between those angles.

As demonstrated by the above examples, multivariate outlier removal considers total multidimensional distance without considering the univariate distances of individual features. It is therefore important to remove univariate and multivariate outliers from your data with separate procedures. If you input a 20 dimensional vector with 19 scalar components close to their respective medians and one scalar component 4 standard deviations away from its median, then the function $-{\sum }_{j}log{(F}^{j }({{X}_{i}}^{j}))$ could fail to discern the scalar outlier in the 20th component because the sum of univariate distances wouldn’t be much different than what you expect by chance. ABOD will also suffer from this problem with 3 or higher dimensional data because the computed angles reside within two-dimensional slices of a higher dimensional space. This means that any single dimension in which one ${{X}_{i}}^{j}$ is a univariate outlier contributes minimally to each angular distance with ${{X}_{i}}^{j}$ as the apex. Points that are more distant from the distribution in all other dimensions by chance could have greater outlier scores.

We demonstrate that the above conjecture is true in Table 1 below. Data ($D$) was simulated from a standard 20 dimensional normal distribution and also a 20 dimensional uniform distribution. Column-wise univariate outliers were simulated by randomly replacing 1% of all scalar values with uniformly selected values from $[\pm max(abs(D)) , \pm (max(abs(D)) + 0.5)]$, thereby ensuring that all univariate outliers are more extreme than all non-outlier scalar values. We then computed the percentage of rows containing univariate outliers that PyOD’s multivariate outlier removal algorithms have assigned top outliership scores. Each multivariate outlier removal algorithm was allowed to remove as many top outliership scores as there were outliers, so the true positive rate (TPR) is the percentage of rows with scalar outliers that were assigned a top multivariate outliership score. Note that any univariate outlier removal algorithm would have a 100% TPR under these circumstances.

**Table 1:** Multivariate outlier removal via PyOD’s algorithms cannot reliably identify data rows that contain a single univariate outlier. Since all of the scalar outliers were simulated to be more extreme than the most extreme non-outlying scalar value across all features, most univariate outlier removal algorithms would have a 100% TPR in this test because they transform scalars to outliership scores monotonically

Model name	Normal TPR	Uniform TPR
ECOD	0.15519	0.15579
COPOD	0.14678	0.13375
KDE	0.84530	0.08795
Sampling	0.72556	0.04861
PCA	0.90761	0.09117
MCD	0.91285	0.09237
OCSVM	0.91123	0.09197
LOF	0.81315	0.10645
COF	0.61499	0.08274
CBLOF	0.88351	0.07951
HBOS	0.04058	0.11448
KNN	0.87467	0.10404
ABOD	0.37622	0.05142
LODA	0.38063	0.05584
SUOD	0.24724	0.12328
VAE	0.91324	0.09037
SO_GAAL	0.02852	0.01365
DeepSVDD	0.15032	0.00081
INNE	0.84045	0.09600
FB	0.76853	0.15307
AutoEncoder	0.91324	0.08997

Table 1 shows that several different multivariate outliership score transformations fail to detect at least 8.7% of rows containing outliers from the simulated 20 dimensional normal data points, and they fail to detect at least 84.4% of such rows from 20 dimensional uniform data points. This performance disparity between the two distributions is expected because the uniform distribution has a higher probability of drawing data rows that are extreme in most dimensions by chance, which means that multivariate outlier detection will invariably assign a greater number of non-outliers particularly high outliership scores by chance.

Multivariate outlier detection can only notice when a single point has extreme values in most or all of its dimensions. An example where multivariate outlier detection would outperform univariate outlier detection is if a datapoint (presumably) from a standard normal distribution equaled 2.9 in all 20 dimensions. Each dimension could reasonably be that extreme individually, but all 20 of them being so extreme is profoundly unlikely to occur by chance. Multivariate outlier removal would rightly classify this datapoint as an outlier, and univariate outlier removal would not. Since the majority of PyOD’s multivariate outlier detection algorithms neither accept one dimensional input nor fit models to their outliership score distributions, these results demonstrate a specific and unmet need for univariate outlier removal algorithms.

### IQR based methods

IQR based tests model data as a normal distribution that was transformed in some way. The most basic IQR test is very popular, even though it inflexibly assumes normality and gives wrong results when this assumption is violated. Let ${p}_{m}$, $QN$ and $IQR$ refer to the ${m}^{th}$ percentile, the ${N}^{th}$ quartile, and $Q3-Q1$ respectively. The IQR test asserts that lower and upper outliership cutoffs exist at ${p}_{0.35}\approx Q1 -1.5IQR$ and ${p}_{99.65} \approx Q3+1.5IQR$ respectively. This outliership test can be explained by substituting in the normal distribution’s approximate quartile values, $Q1 \approx -0.675\sigma .$ and $Q3 \approx 0.675\sigma .$ The expressions then condense to ${p}_{0.35}\approx -2.7\sigma$ and ${p}_{99..65}\approx 2.7\sigma$, which is false if the distribution is not normal. Therefore, if the goal is to remove points outside of the percentile range $\left[{p}_{0.35}{, p}_{99.65}\right]$ of a fitted model distribution because those points are likely to be outliers, then the IQR test is wrong when the underlying distribution is not normal. A desire to improve upon this method has led to many numerical corrections. For example, Hubert and Vanderviere adjust the standard IQR cutoffs as $[Q1 - 1.5{e}^{(aMC)}IQR, Q3 + 1.5{e}^{(bMC)}IQR]$, where MC is the medcouple and (a =  − 3.79, b = 3.87) are empirically fitted coefficients [7]. There are also methods that reduce skew by iteratively removing outliers and refitting the model’s outlier bounds [8, 9], though they still assume that the input outliership scores are normally distributed.

We decided to base our own method upon a generalized IQR based method created by Verardi and Vermandele [10]. Each scalar datapoint is transformed by subtracting from the median and dividing by the inner quartile range of the datapoint’s side, which is defined as the asymmetrical outlyingness (ASO) in Sect. 4.2 of [10]. Then the ASOs are probit-transformed, which results in a normal distribution if the ASO is uniformly distributed; otherwise, it results in an unknown transformation of a normal distribution that introduces both skew and tail heaviness [10]. The authors of [10] compare such ASO-probit transformed normal distributions to the four parameter Tukey-gh distribution, which also transforms normal distributions in a way that introduces skew and kurtosis with reasonable generality. Taylor series analysis of the Tukey-gh transformation’s multiplicative components show that the g and h parameters independently control the transformed distribution’s odd and even moments respectively [11, 12]. Giving each polynomial component an independent regression coefficient could theoretically improve model flexibility until full generality is reached [11, 12], but this appears to be unnecessary most of the time [10].

## Methods

The inability of [10] to handle multimodality is one of its few notable weaknesses, though this is discussed by the authors only briefly. To examine the consequences of ignoring this weakness, we simulated outliers on 50 gaussian mixture tri-modal distributions with fixed intermodal distances of 5, 5.6, …, 33. We show that the proportion of outliers detected decreases steadily as the gap between peaks increases. Our results demonstrate that this occurs simply because [10] cannot correctly model multimodal data. More serious problems may occur in real mixture model data with distributions of different sizes and moments, which highlights the need to model multimodality.

Despite the ASO-probit transformation’s attempt to account for skew, we show that it fails to detect outliers from the thin tailed side of sufficiently skewed distributions. It is simply the case that outliers on the thin tailed side don’t score as highly as non-outliers on the fat tailed side, which necessitates an alternative way to account for skew. We demonstrate this problem by simulating outliers on fifty Tukey-gh distributions with h = 0 and g = 0.015, 0.03, …, 0.75. We show that the proportion of outliers detected decreases steadily with increasing skew until the TPR remains near 0.5 for distributions with greater skew because the transformation itself fails to capture outlyingness in the thin tail. Our results demonstrate how the ASO-probit transformation alone places half of all outliers (i.e. all of the light tail’s outliers) noticeably before the 99.3rd percentile cutoff.

Additionally, although the authors of [10] show one figure where their model appears to fit an exponential distribution, we have found that it usually fails to fit monotonic distributions. We tested this apparent discrepancy by simulating outliers for fifty monotonic distributions that were created by transforming standard exponential random variables $X$ via ${X}_{transformed} = {X}^{a}$ for a = 1.03, 1.06, …, 2.5. We show that the ASO-probit transformation does not smoothly transform such exponentially shaped distributions, and that the TPR decreases to 0 as a increases. This seems to happen because [10] simply cannot fit certain monotonic distributions.

We then compare the efficacy of STAR_outliers to that of other algorithms (Table 2) on different simulated distributions (Table 3). We simulated 100,000 datapoints from 10 types of distributions, each of which was simulated for 10 different parameter values, for a total of 100 distributions. We randomly replaced three hundred datapoints from each distribution with outliers to measure each algorithm’s efficacy at detecting and removing outliers. For one sided distributions, each outlier was drawn from ${p}_{99.5} + \epsilon : \epsilon \sim Uniform(0.5\sigma , 2\sigma )$, where $\sigma$ is the respective distribution’s standard deviation. For two sided distributions, half of all selected values were converted to ${p}_{99.75} + \epsilon$, while the other half were converted to ${p}_{0.25} - \epsilon$.

**Table 2:** A list of algorithms compared to STAR_outliers. Algorithms are detailed in the figure generation repository

Algorithm	Algorithm type	Description
IF	IF	IF out of the box model
IF-calibrated	IF	IF calibrated to remove as many outliers as STAR_outliers
STAR	STAR_outliers	STAR_outliers
[3] (*p* = 90)	[3]	[3] using percentiles 90 and 10 to estimate Tukey-gh parameters
[3] (*p* = 91)	[3]	[3] using percentiles 91 and 9 to estimate Tukey-gh parameters
[3] (*p* = 92)	[3]	[3] using percentiles 92 and 8 to estimate Tukey-gh parameters
[3] (*p* = 93)	[3]	[3] using percentiles 93 and 7 to estimate Tukey-gh parameters
[3] (*p* = 94)	[3]	[3] using percentiles 94 and 6 to estimate Tukey-gh parameters
[3] (*p* = 95)	[3]	[3] using percentiles 95 and 5 to estimate Tukey-gh parameters
[3] (*p* = 96)	[3]	[3] using percentiles 96 and 4 to estimate Tukey-gh parameters
[3] (*p* = 97)	[3]	[3] using percentiles 97 and 3 to estimate Tukey-gh parameters
[3] (*p* = 98)	[3]	[3] using percentiles 98 and 2 to estimate Tukey-gh parameters
[3] (*p* = 99)	[3]	[3] using percentiles 99 and 1 to estimate Tukey-gh parameters
T2	[9]	T2 with 2 iterations
T2_yj	[9]	T2 with 2 iterations for yj transformed data
T3	[9]	T2 with 3 iterations
T3_yj	[9]	T2 with 3 iterations for yj transformed data
T4	[9]	T2 with 4 iterations
T4_yj	[9]	T2 with 4 iterations for yj transformed data
3SD	normal	standard 3SD cutoff
3SD_yj	normal	3SD cutoff for yj transformed data
IQR	normal	standard IQR cutoff
IQR_yj	normal	standard IQR cutoff for yj transformed data
MAD	normal	standard MAD cutoff
MAD_yj	normal	standard MAD cutoff for yj transformed data

**Table 3:** A list of distributions with simulated outliers analyzed by the algorithms in Table 2. Nonstandard distributions include uniform distributions, triangular distributions, multimodal normal distributions, and different mixtures thereof. They are defined in step1 of the figure generation repository

distribution Type	Description
lognormal	lognormal distribution: 10 spread parameter values
exponential	exponential random variables drawn and then raised to a power: 10 power values
power	power distribution: 10 power values
poisson	poisson distribution: 10 parameter values
negative binomial	negative binomial distribution: 10 success probabilities
Tukey-g	tukey-gh distribution: 10 g values, h = 0
Tukey-h	tukey-gh distribution: 10 h values, g = 0
Tukey-gh	tukey-gh distribution: 10 values, g = h
beta	beta distribution: 10 beta values, alpha = 2
non-standard	various non-smooth and/or multimodal distributions: 10 different shapes

For each type of algorithm, the F1 scores for algorithms of that type were compared, and the algorithm with the highest F1 score was selected for followup testing (step 7 in the figure generation repository). TPRs and FPRs (false positive rates) were determined for the selected algorithms’ performances on all 100 distributions, which is possible because the ground truth is known. We have observed that the IF algorithm’s default outliership score model in PyOD usually has an excessively high false positive rate (FPR). We compensated for this with an alternative method that simply removes as many outliers as STAR_outliers detected in order of decreasing IF outliership score. This method (IF-calibrated) gives the IF method an equal number of opportunities to remove the correct outliers as STAR_outliers, thus ensuring that their comparison is fair.

Finally, we compared the selected algorithms’ performances on the real 2018 NHANES Demographics, Dietary, Laboratory, Examination and Questionnaire data subsets [13]. In this test, an ideal outlier removal algorithm would remove an average of 0.7% of all data points plus any outliers in the data. Assuming that the data contains relatively few outliers, we assume that about 0.7% of all datapoints should be removed from every univariate distribution. Therefore, for each outlier removal algorithm, we compute the mean absolute difference from 0.7% of outliers removed across 208 features in NHANES dataset that have at least 10 unique numerical (i.e. noncategorical) values. Note that we cannot include the IF-calibrated method in this test because it relies on STAR_outliers to remove the expected number of datapoints.

## Implementation

**Fig. 1:**
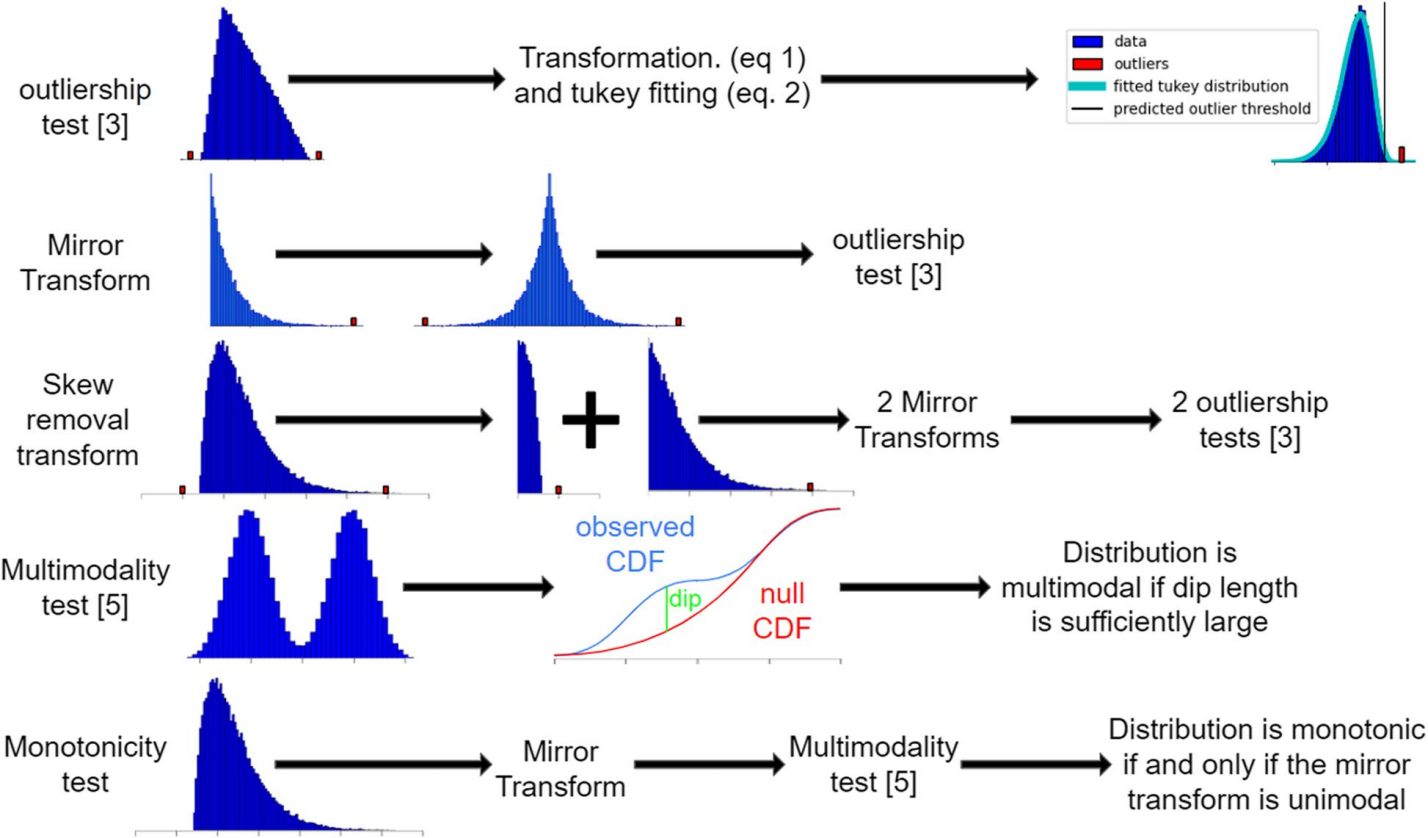
A diagram of tests and transforms used by STAR_outliers

**Fig. 2:**
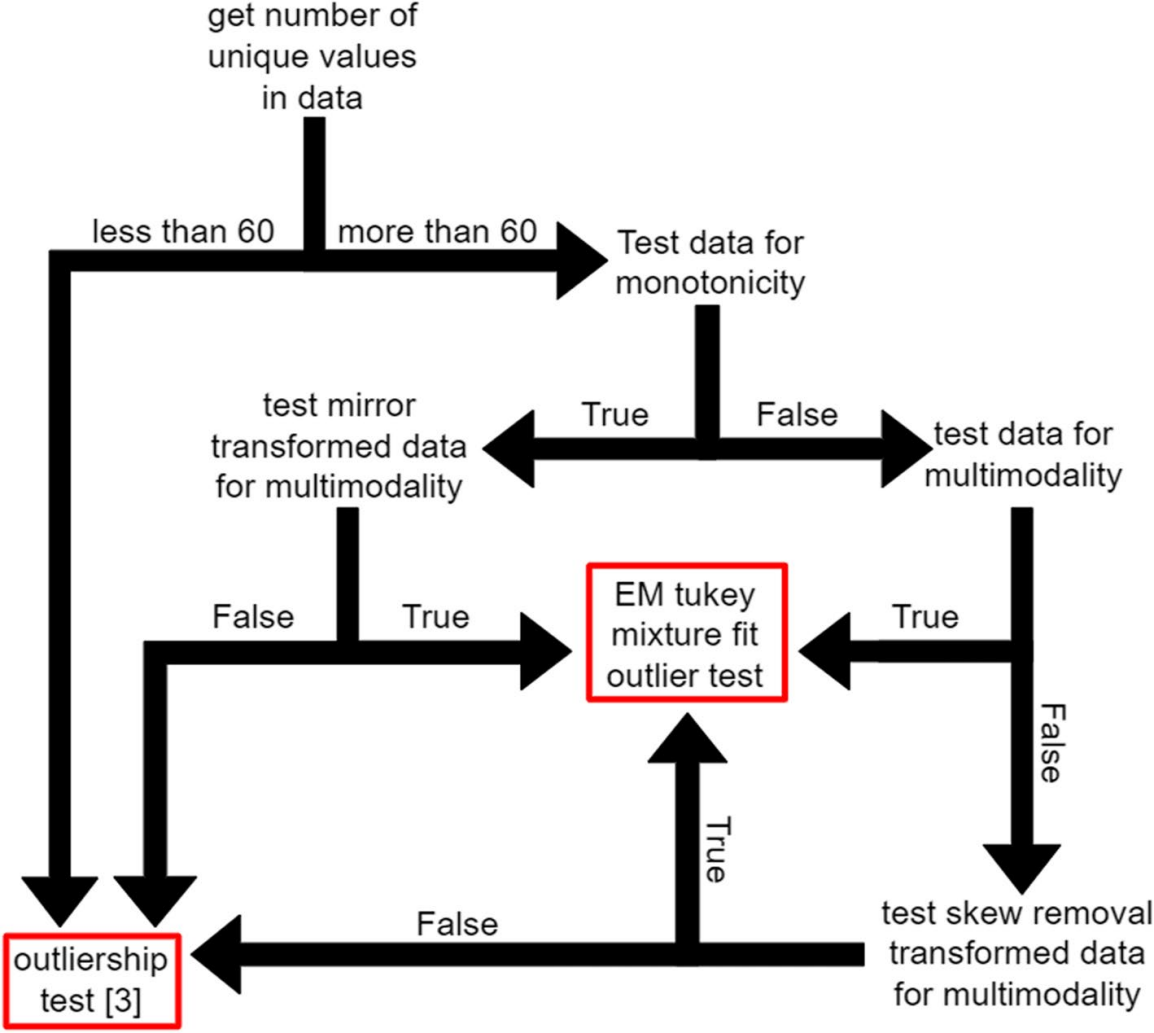
The procedure that STAR_outliers uses to test for outliers

Our objective was to implement an IQR-based algorithm that can robustly remove outliers in an unsupervised manner from most distribution types, including discrete, multimodal, skewed, and monotonic distributions. We do this by modifying an existing algorithm [10] with several other well-tested algorithms to correctly compensate for the weakness described in the methods. Figure 1 displays summary descriptions of these algorithms, and their applications are detailed in Fig. 2. Prior to removing outliers with [10] at any point, both the original and transformed distributions with at least 60 unique values are tested for multimodality with Hartigan's dip test [14]. Briefly, the empirical CDF is compared to the non-intersecting unimodal CDF with the lowest maximum vertical distance. This distance is the test statistic, which is bootstrapped by resampling data from the unimodal CDF 30 times to compute 30 null test statistics. The distribution is determined to be multimodal if the real statistic exceeds all test statistics and exceeds 0.0001, which corresponds to multimodality that is barely visible upon inspection.

If the original distribution is not determined to be multimodal, then STAR_outliers concatenates the broad sides of the distribution and its mirror image (a mirror transform) and tests the resulting concatenation for multimodality. Mirror transformed monotonic distributions will test negative for multimodality, while non-monotonic distributions will test positive. Since [10] is easily able to detect outliers in mirrored monotonic distributions such as the triangular and the laplace, STAR_outliers applies [10] to mirror transformed monotonic distributions. If the distribution is determined not to be monotonic, then skewness is handled simply by splitting each unimodal distribution at the peak and applying the previous mirror image concatenation procedure to each side of the monotonic distribution. As insurance against the possibility that discrete random variables would bias the quantile based Tukey-gh parameter estimates of [10], Tukey-gh parameters are estimated from 100 percentiles with percentile regression [15].

If the original or the transformed distribution is determined to be multimodal, then we use an approximate EM algorithm to fit a bimodal Tukey-gh mixture model to the transformed distribution. The mixture could include more modes, but data with three or more modes is rare, which makes such a modification more likely to overfit discreteness than to correctly account for higher modality. The E step uses the most recent Tukey-gh parameter values to update the probability that each transformed data value belongs to each Tukey-gh in the mixture model. The M step updates the mixture model’s Tukey-gh parameters by stochastically assigning each transformed data value to one of the Tukey-gh distributions in accordance with their respective probabilities of drawing that value. Then it estimates the Tukey-gh distributions’ parameters with a slightly modified version of [15] from the assigned data values. A derivation that this procedure normally converges to standard EM in the limit of large data is in the Additional file 1. Note that our modifications of [15] slightly bias the g and h parameters toward 0, which is useful to prevent our method from overfitting discrete data. They also upweight the importance of fitting high percentile datapoints, which ensures that the model’s tail fits well in the event of an imperfect overall fit to the real data.

## Results

**Fig. 3:**
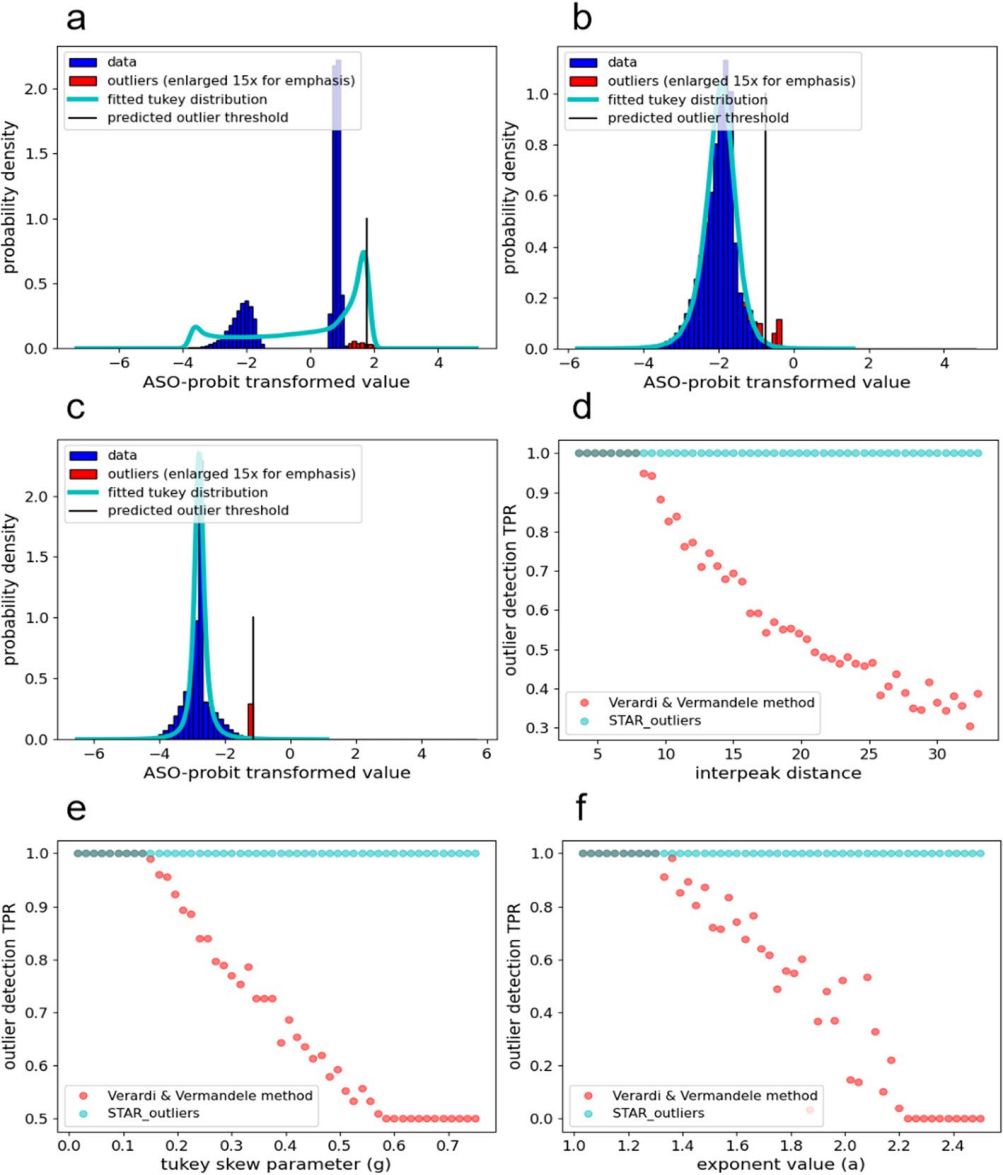
Each sub-figure characterizes a specific flaw in how [10] handles skew, monotonicity, or multimodality

The first half of Fig. 3 qualitatively demonstrates the problem with multimodality (Fig. 3a), skewness (Fig. 3b), and monotonicity (Fig. 3c). Distributions are of ASO-probit transformed values, and the outlier bin sizes are increased 15 fold for ease of viewing. The second half of Fig. 3 quantifies how STAR_outliers improves [10] when handling Multimodality, skew, and monotonicity. Figure 3d shows that, without STAR_outlier’s EM fitting algorithm, the outlier detection TPR steadily decreases as the interpeak distance increases. Figure 3e shows that the proportion of outliers caught by [10] steadily decreases as the skew increases. Since all missed outliers are in the distributions’ light tails, this demonstrates that the ASO fails to correctly account for high amounts of skew, and that skewed distributions’ sides need to be analyzed separately, such as with our mirror transform. Figure 3f shows that [10] simply fails to smoothly transform most monotonic distributions, which necessitates our novel monotonicity test and corresponding mirror transformation. Figure 3 therefore demonstrates the need for the specific improvements that STAR_outliers provides.

**Fig. 4:**
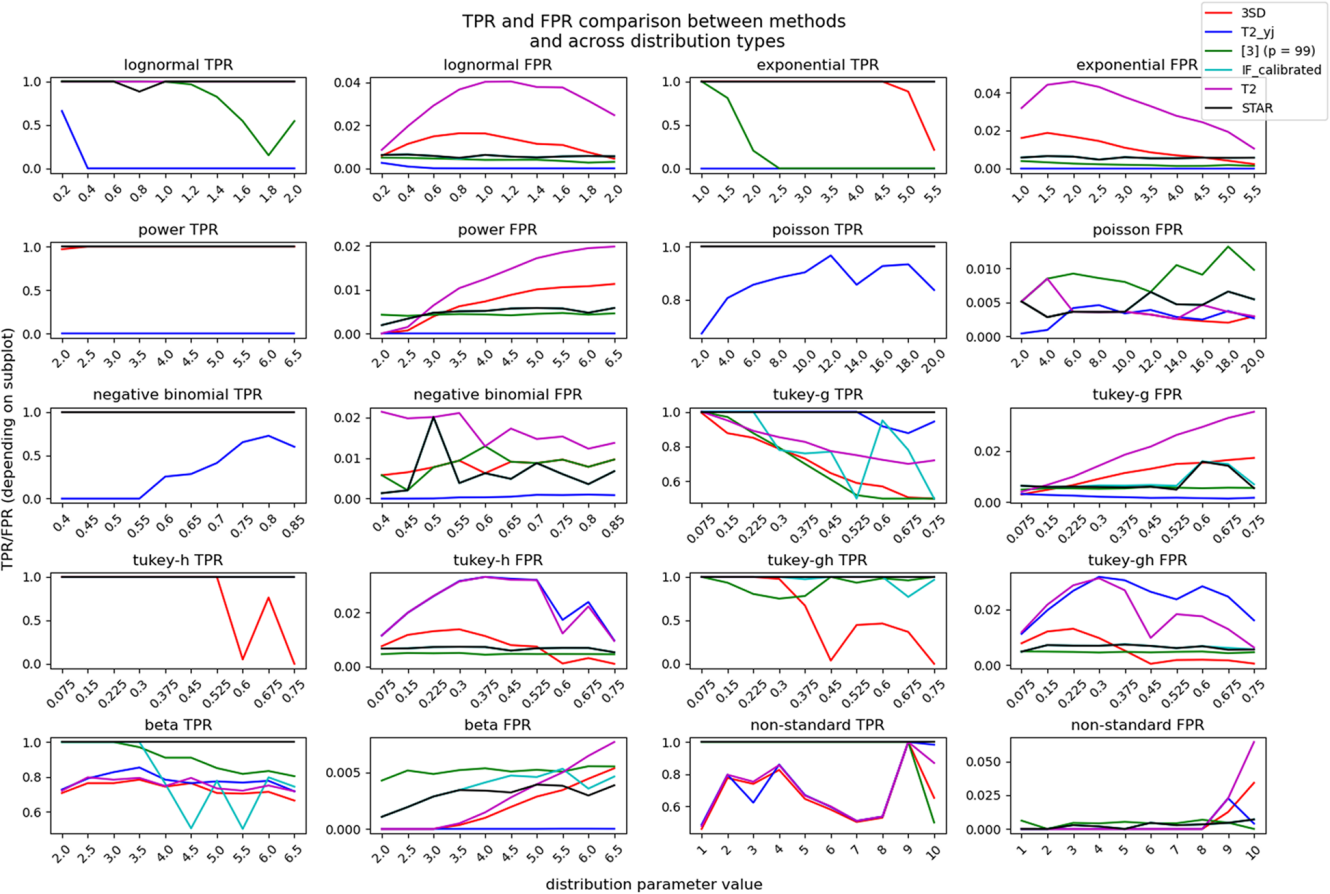
Numbers 1–10 in the non-standard distributions’ x axes refer to simulated distributions 91–100 in step1 of the figure generation repository

Figure 4 shows that STAR_outliers removes outliers more effectively than the best method of each type. We also included the T2 method on data that is not yeo-johnson transformed for good measure. In general, all of the outlier removal methods demonstrate low TPR for some parameter values in at least two distribution types, and many of them also have high FPRs for other distribution types. Notice that varying an outlier removal method’s parameters is unlikely to fix this problem. If the 3SD method is changed to a 2.5SD method to improve the TPR for certain distributions, then that will worsen the FPR for other distributions, which makes tinkering with such parameters less effective than modeling distributions correctly in the first place. The IF-calibrated method performs most comperably to STAR_outliers, demonstrating that it could effectively remove univariate outliers if it would fit a better model to the outliership scores. Even so, the IF-calibrated method also underperforms on distributions with high skew and normal kurtosis in a manner similar to [10], as the IF-calibrated method also fails to detect most outliers in the most skewed Tukey-g distributions’ short tails. Note that increasing both the kurtosis and the skew appears to dampen this effect, indicating that it is caused by relative differences in tail heaviness. This further highlights the importance of STAR_outlier’s skew removal transform.

**Fig. 5:**
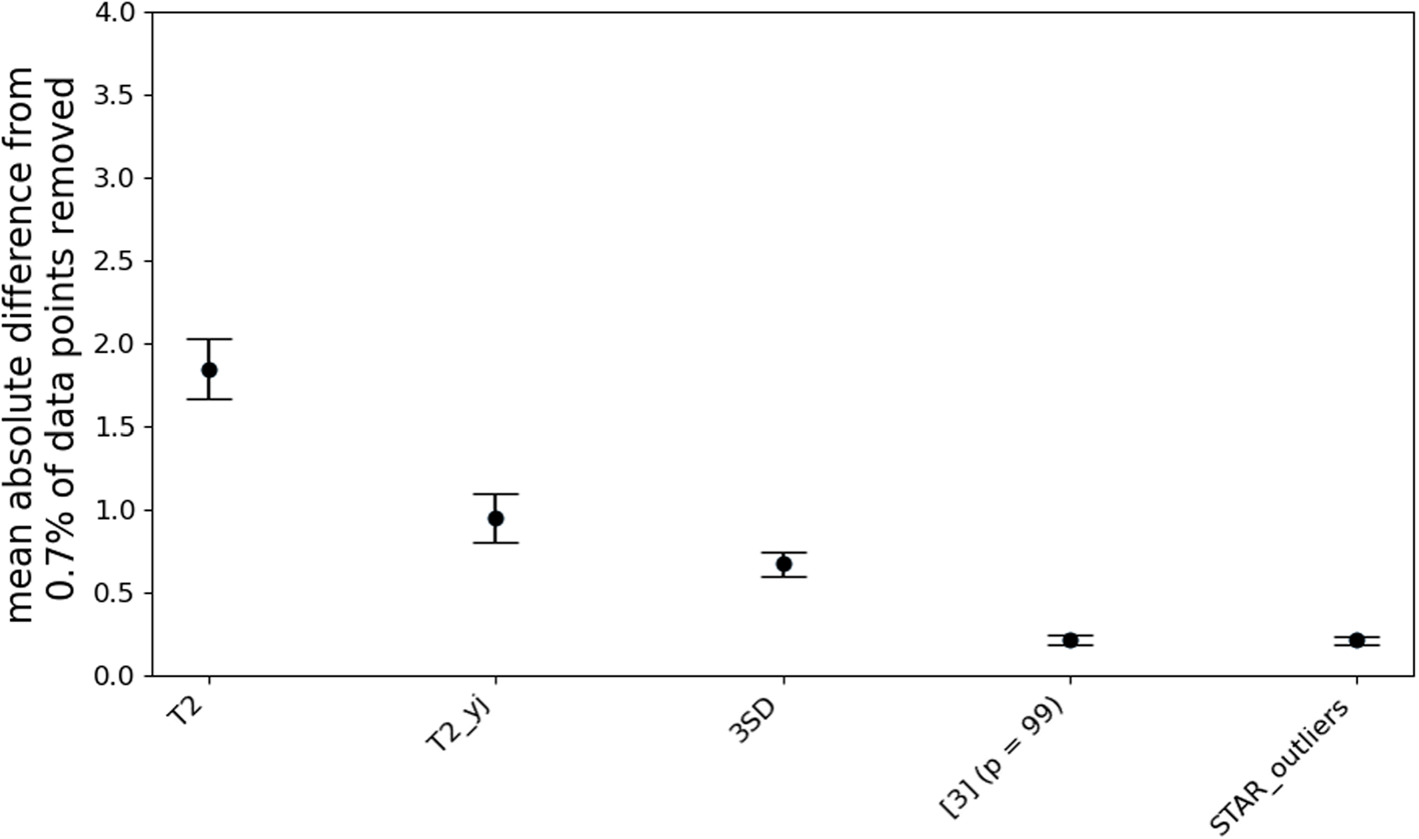
STAR_outliers consistently removes closer to 0.7% of a dataset than the other algorithms (i.e. the mean absolute difference from 0.7% is closest to 0), except for [10] after setting *p* = 99 against its authors’ recommendations. Despite removing the correct number of points, [10] still fails to account for skew, bimodality, and monotonicity, while STAR_outliers does all of these things correctly

Figure 5 shows that STAR_outliers and [10] (with *p* = 0.99) both remove significantly closer to 0.7 percent of the datapoints across NHANES dataset features than any of the best outlier removal algorithm types, indicating that their Tukey-gh based model of ASO-probit transformed data provides a superior fit to most distributions. While STAR_outliers and [10] appear to fit real transformed data distributions equally well when p is set to equal 0.99, we have also shown that [10] transforms outliers in the short tail into lower outliership scores than non-outliers in the long tail. Such inaccuracies cannot be observed in Fig. 5 because the outlier statuses are unknown, which makes STAR_outliers the most accurate outlier removal procedure. We have also demonstrated that [10] fits exponential and multimodal distributions poorly, meaning that these results for [10] cannot be expected to generalize to other datasets. Finally, we could not find an easily installable algorithm for [10], which leaves STAR_outliers as a unique and ready-to-use outlier removal algorithm that can correctly handle the vast majority of distribution shapes.

## Conclusions

We have demonstrated an easily implemented python package that is objectively superior to other modern unsupervised univariate outlier removal programs. Given recent interest in detecting multi-dimensional outliers, it's worth noting that modifying STAR_outliers to detect multidimensional outliers would be relatively straightforward by adhering to the generalization described in [10]. Alternatively, one could compute a distribution of multivariate outliership scores with an existing algorithm like COPOD, and use STAR outliers to fit that distribution for the purpose of outlier removal.

### Supplementary Information

**Additional file 1.** Stochastic EM Supplementary Derivation.

## Data Availability

The raw NHANES datasets analysed during the current study are available at https://wwwn.cdc.gov/nchs/nhanes/continuousnhanes/default.aspx?BeginYear=2017 at the links under the “Data, Documentation, Codebooks” heading. The compiled NHANES datasets, code for creating simulated datasets, and code for generating the paper’s figures are in the figure_and_table_generation file at https://github.com/EpistasisLab/STAR_outliers. Project name: STAR_outliers, Project home page: https://github.com/EpistasisLab/STAR_outliers, Figure generation repository: https://github.com/EpistasisLab/STAR_outliers_figure_and_table_generation, Operating systems: Ubuntu (Linux), Mac, Windows, Programming language: Python (versions 3.6–3.9), License: STAR_outliers.

## Abbreviations

IF: Isolation forest
IQR: Interquartile range
TPR: True positive rate
FPR: False positive rate
STAR_outliers: Skew and tail-heaviness adjusted removal of outliers
NHANES: National Health and Nutrition Examination Survey
YJ: Yeo-Johnson (referring to the normality transformation)
