# Supplementary material for: STAR_outliers: a python package that separates univariate outliers from non-normal distributions
Source: BioData Min. 2023 Sep 4;16:25. doi: 10.1186/s13040-023-00342-0 (PMC10476292; doi:10.1186/s13040-023-00342-0)
Supplement: Supplementary file 1 — Additional file 1. Stochastic EM Supplementary Derivation. [file 13040_2023_342_MOESM1_ESM.pdf]

Stochastic EM Supplementary Derivation

1 background

The goal of the maximization step in EM is to minimize the following sum with respect to θ and Z.

$$\frac{1}{N}\sum_{i}^{N}\sum_{k}^{K} q(Z_i = k|X_i = x_i, \theta) log(p(Z_i = k, X_i = x_i|\theta))$$

It would be convenient to sample one value (call it z_i) for each Z_i from each $q(Z_i = z|X_i = x_i)$ and then to substitute each $q(Z_i = z|X_i = x_i)$ with $I(Z_i = z)$, where I is the identity function. This gives each x_i a randomly sampled hard assignment of z_i for the maximization update, which reduces the first sum to the following:

$$\frac{1}{N}\sum_{i}^{N} log(p(Z_i = z_i, X_i = x_i|\theta))$$

This approximation allows θ to be updated with standard MLE estimates as opposed to the usual modified pseudo-MLE estimate for EM, which is useful if p has no explicit functional form. We justify this approximation by proving the following:

$$\frac{1}{N}\sum_{i}^{N} log(p(Z_i = z_i, X_i = x_i|\theta)) \xrightarrow{a.s.} \frac{1}{N}\sum_{i}^{N}\sum_{k}^{K} q(Z_i = k|X_i = x_i, \theta) log(p(Z_i = k, X_i = x_i|\theta))$$

2 proof

each z_i is drawn from $q(Z_i = z|X_i = x_i, \theta)$ as follows:

$$q(Z_i = z|X_i = x_i, \theta) = \frac{p(X_i = x|Z_i = z, \theta)p(Z_i = z|\theta)}{\sum_k^K p(X_i = x|Z_i = k, \theta)p(Z_i = k|\theta)}$$

let $p(Z_i = z|\theta) = f_z$ be the fraction of x_i assigned to group z in the previous group update.

$$q(Z_i = z|X_i = x_i, \theta) = \frac{p(X_i = x|Z_i = z, \theta)f_z}{\sum_k^K p(X_i = x|Z_i = k, \theta)f_k}$$

Allow each z_i value to be drawn from $q(Z_i = z|X_i = x_i, \theta)$:

$$E_i\Big[log(p(Z_i = z_i, X_i = x_i|\theta))\Big]$$

$$= E_{x,z}\Big[log(p(Z_i = z, X_i = x|\theta))\Big]$$

$$= E_x\Big[E_{z|x}\Big[log(p(Z_i = z, X_i = x|\theta))\Big]\Big]$$

$$= E_x\Big[\sum_k^K q(Z_i = k|X_i = x, \theta)log(p(Z_i = k, X_i = x|\theta))\Big]$$

$$= E_i\Big[\sum_k^K q(Z_i = k|X_i = x_i, \theta)log(p(Z_i = k, X_i = x_i|\theta))\Big]$$

Thus:

$$E_i\Big[log(p(Z_i = z_i, X_i = x_i|\theta))] = E_i\Big[\sum_k^K q(Z_i = k|X_i = x_i, \theta)log(p(Z_i = k, X_i = x_i|\theta))\Big]$$

It follows from the law of large numbers that:

$$\frac{1}{N}\sum_i^N log(p(Z_i = z_i, X_i = x_i|\theta)) \xrightarrow{a.s.} \frac{1}{N}\sum_i^N\sum_k^K q(Z_i = k|X_i = x_i, \theta)log(p(Z_i = k, X_i = x_i|\theta))$$

3 procedure

Let $Z_{ij} \in \{1, 2\}$ refer to which tukey distribution in the mixture model that x_i was stochastically assigned to at the jth iteration. Refer to the parameters for both tukey distributions as $\theta_j = \{A_{1j}, A_{2j}, B_{1j}, B_{2j}, g_{1j}, g_{2j}, h_{1j}, h_{2j}\}$ at the jth iteration. Let T refer to the four parameter tukey distribution. The EM algorithm above is described as follows:

E step:

$$q(Z_{ij} = z_{i(j+1)}|X_i = x_i, \theta_j) = \frac{p(X_i = x_i|Z_{ij} = z_{ij}, \theta_j)f_{z(j)}}{\sum_k p(X_i = x_i|Z_{ij} = k, \theta_j)f_{k(j)}}$$

$$= \frac{T(X_i = x_i|Z_{ij} = z_{ij}, A_{zj}, B_{zj}, g_{zj}, h_{zj})f_{z(j)}}{T(X_i = x_i|Z_{ij} = 1, A_{1j}, B_{1j}, g_{1j}, h_{1j})f_{1(j)} + T(X_i = x_i|Z_{ij} = 2, A_{2j}, B_{2j}, g_{2j}, h_{2j})f_{2(j)}}$$

M step:

Draw $z_{i(j+1)}$ from $q(Z_{ij} = z_{i(j+1)}|X_i = x_i, \theta_j)$ for each x_i.Assign each x_i to the tukey distribution indicated by $z_{i(j+1)}$:

$$LL(\theta_j) = \sum_i^N log(p(Z_{i(j+1)} = z_{i(j+1)}, X_i = x_i|\theta_j))$$

$$= \sum_{i}^{N} log(p(X_i = x_i | Z_{i(j+1)} = z_{i(j+1)}, \theta_j) p(Z_{i(j+1)} = z_{i(j+1)} | \theta_j))$$

$$= \sum_{i}^{N} log(p(X_i = x_i | Z_{i(j+1)} = z_{i(j+1)}, \theta_j)) + \sum_{i}^{N} log(p(Z_i = z_{i(j+1)} | \theta_j))$$

Let $p(Z_i = z_{i(j+1)} | \theta_j) = f_{z(j+1)(i)}$, which is fixed and independent of θ_j for all i during the $(j+1)^{th}$ M step. Let N_1 and N_2 refer to the number of x_i that were assigned to groups $z = 1$ and $z = 2$ respectively

$$LL(\theta_j) = \sum_{i}^{N_1} log(T(X_i = x_i | Z_{i(j+1)} = 1, A_1, B_1, g_1, h_1))$$

$$+ \sum_{i}^{N_2} log(T(X_i = x_i | Z_{i(j+1)} = 2, A_2, B_2, g_2, h_2))$$

$$+ \sum_{i}^{N} log(f_{z(j+1)(i)})$$

The third term can be ignored because it is constant with respect to θ_j. Then we use a modified version of [4] with an L2 penalty on the g and h parameters to estimate $\{A_{1(j+1)}, B_{1(j+1)}, g_{1(j+1)}, h_{1(j+1)}\}$ from $\{x_i : z_{i(j+1)} = 1\}$, and we separately estimate $\{A_{2(j+1)}, B_{2(j+1)}, g_{2(j+1)}, h_{2(j+1)}\}$ from $\{x_i : z_{i(j+1)} = 2\}$. This would converge to True EM if we used the consistent tukey parameter estimator in [4]. However, we have observed quantile regression to occasionally produce a non-smooth distribution that has a large negative h parameter, particularly when the underlying data is discrete. The L2 penalty prevents this from happening. We also upweight the error terms on quantiles that are closer to 100 because all original outliers become outliers with respect to the transformed distribution's right tail.

Compute $LL(\theta_{j+1}) = \sum_{i}^{N} log(p(Z_{i(j+1)} = z_{i(j+1)}, X_i = x_i | \theta_{j+1}))$. Repeat E and M steps from $j+1$ if $LL(\theta_{j+1}) > LL(\theta_j)$. Otherwise, rerun steps from j. Repeat until 20 consecutive non-improvements occur. We repeat this procedure for four different parameter initializations with and without another penalty that encourages tukey center separation. The parameters with the highest log likelihood are selected for the final tukey model.
